# Metabolomic profiling in small vessel disease identifies multiple associations with disease severity

**DOI:** 10.1093/brain/awac041

**Published:** 2022-02-08

**Authors:** Eric L Harshfield, Caroline J Sands, Anil M Tuladhar, Frank Erik de Leeuw, Matthew R Lewis, Hugh S Markus

**Affiliations:** Stroke Research Group, Department of Clinical Neurosciences, University of Cambridge, Cambridge CB2 0QQ, UK; National Phenome Centre, Department of Metabolism, Digestion and Reproduction, Imperial College London, London SW7 2AZ, UK; Department of Neurology, Donders Center for Medical Neuroscience, Radboud University Nijmegen Medical Center, 6500 HB Nijmegen, The Netherlands; Department of Neurology, Donders Center for Medical Neuroscience, Radboud University Nijmegen Medical Center, 6500 HB Nijmegen, The Netherlands; National Phenome Centre, Department of Metabolism, Digestion and Reproduction, Imperial College London, London SW7 2AZ, UK; Stroke Research Group, Department of Clinical Neurosciences, University of Cambridge, Cambridge CB2 0QQ, UK

**Keywords:** metabolomics, small vessel disease, stroke, dementia, cognition

## Abstract

Cerebral small vessel disease is a major cause of vascular cognitive impairment and dementia. There are few treatments, largely reflecting limited understanding of the underlying pathophysiology. Metabolomics can be used to identify novel risk factors to better understand pathogenesis and to predict disease progression and severity.

We analysed data from 624 patients with symptomatic cerebral small vessel disease from two prospective cohort studies. Serum samples were collected at baseline and patients underwent MRI scans and cognitive testing at regular intervals with up to 14 years of follow-up. Using ultra-performance liquid chromatography–mass spectrometry and nuclear magnetic resonance spectroscopy, we obtained metabolic and lipidomic profiles from 369 annotated metabolites and 54 764 unannotated features and examined their association with respect to disease severity, assessed using MRI small vessel disease markers, cognition and future risk of all-cause dementia.

Our analysis identified 28 metabolites that were significantly associated with small vessel disease imaging markers and cognition. Decreased levels of multiple glycerophospholipids and sphingolipids were associated with increased small vessel disease load as evidenced by higher white matter hyperintensity volume, lower mean diffusivity normalized peak height, greater brain atrophy and impaired cognition. Higher levels of creatine, FA(18:2(OH)) and SM(d18:2/24:1) were associated with increased lacune count, higher white matter hyperintensity volume and impaired cognition. Lower baseline levels of carnitines and creatinine were associated with higher annualized change in peak width of skeletonized mean diffusivity, and 25 metabolites, including lipoprotein subclasses, amino acids and xenobiotics, were associated with future dementia incidence.

Our results show multiple distinct metabolic signatures that are associated with imaging markers of small vessel disease, cognition and conversion to dementia. Further research should assess causality and the use of metabolomic screening to improve the ability to predict future disease severity and dementia risk in small vessel disease. The metabolomic profiles may also provide novel insights into disease pathogenesis and help identify novel treatment approaches.

## Teaser

Harshfield *et al.* analyse metabolomic profiles from cerebral small vessel disease (SVD) patients, identifying 28 metabolites associated with SVD imaging markers and cognition, and 25 metabolites associated with future risk of dementia. The profiles may provide insights into disease pathogenesis and help inform new treatments.

## Introduction

Cerebral small vessel disease (SVD) accounts for a quarter of all ischaemic strokes and is the most common pathology underlying vascular cognitive impairment and dementia.^1^ SVD is characterized by typical radiological features seen on brain MRI including lacunes, white matter hyperintensities (WMH), cerebral microbleeds, diffuse ultrastructural changes that can be detected using diffusion tensor imaging (DTI) and brain atrophy. Despite its importance, there are few effective treatments for delaying disease progression. A major reason for this is limited understanding of the disease pathogenesis. Furthermore, although it is a major cause of dementia, only a proportion of patients with radiological SVD progress to cognitive impairment.^2^ Once effective treatments become available, predicting which patients are at elevated risk will become clinically important, and better markers of disease progression are therefore required.^3^

Metabolomics, the high-throughput identification and quantification of small molecules in biological samples, offers the potential to both identify novel disease mechanisms and develop better predictive markers.^4^ Metabolomics assays surpass standard chemistry techniques for the purposes of comprehensive metabolome measurement^5^ since they are capable of precise analysis of hundreds or even thousands of metabolites.^6^ This allows detailed characterization of metabolic phenotypes, enabling characterization of metabolic arrangements underlying disease pathogenesis, discovery of new therapeutic markers and identification of novel biomarkers to diagnose and monitor disease.^6^ Metabolomics has been applied successfully in a number of cardiovascular and neurological diseases,^7,8^ but there have been few studies in SVD.

Ultra-performance liquid chromatography–mass spectrometry (UPLC–MS) and nuclear magnetic resonance (NMR) spectroscopy are effective analytical techniques for detecting and measuring chemical constituents within blood samples. In this analysis we obtained baseline metabolomics profiles from 624 patients with symptomatic MRI-confirmed SVD and up to 14 years of follow-up. We examined associations between metabolites and disease severity, assessed using both MRI disease markers and cognitive parameters. We also evaluated relationships between metabolites and future risk of all-cause dementia.

## Materials and methods

### Data sources

We analysed individual participant data from two studies involving patients with symptomatic SVD: (i) St George's Cognition and Neuroimaging in Stroke (SCANS), a longitudinal study of cognitive impairment in 121 patients with moderate to severe symptomatic SVD^2,9^; and (ii) the Radboud University Nijmegen Diffusion Tensor and Magnetic Resonance Imaging Cohort (RUN-DMC), a prospective cohort study from the Netherlands of 503 individuals aged between 50 and 85 years with symptomatic SVD.^10^ SCANS participants had multimodal MRI and cognitive tests performed at baseline and at Years 1, 2 and 3, as well as 5-year follow-up for dementia, and RUN-DMC participants had MRI, cognitive, and clinical assessments performed at baseline and at Years 5, 9 and 14, with 14 years of follow-up for dementia. Both studies recorded information from each participant on a range of demographics and vascular risk factors, including sex, age, ethnicity, body mass index, smoking status, diabetes status, systolic and diastolic blood pressure, hypertension status and hypercholesterolaemia status. Follow-up data on dementia incidence was available for all 121 patients from SCANS and 501 patients from RUN-DMC.

In SCANS, SVD was defined as a clinical lacunar stroke syndrome with MRI evidence of an anatomically corresponding lacunar infarct, and with confluent regions of WMH graded ≥2 on the modified Fazekas scale.^2^ Exclusion criteria included if the patient had any stroke mechanism other than SVD (extra or intracranial large artery stenosis >50%, cardioembolic source, non-lacunar subcortical infarcts >1.5 cm in diameter as these are often caused by emboli or cortical infarcts), any cardioembolic cause of stroke diagnosed using Trial of Org 10172 in Acute Stroke Treatment (TOAST) criteria,^11^ or if they had a history of major neurological or psychiatric disorders (with the exception of depression). In RUN-DMC, inclusion criteria were: (i) age between 50 and 85 years; and (ii) cerebral SVD on neuroimaging, defined as the presence of either WMH or lacunes.^10^ Exclusion criteria included the presence of dementia, parkinsonism, an intracranial space occupying lesion, non-SVD related WMH (e.g. multiple sclerosis) or life expectancy of <6 months. Both studies also excluded individuals with known monogenic SVD.

### Metabolomics data

Serum samples collected at baseline from 624 participants from the SCANS and RUN-DMC cohorts were analysed using UPLC–MS and proton ^1^H NMR spectroscopy. Full analytical details, following previously described sample preparation, analytical and quality control (QC) procedures,^12–17^ are provided in the Supplementary material. For each assay, samples were analysed in a randomized order demonstrating no correlation or other relationship with study design variables, precluding any confounding effect of analysis order. To facilitate quality assessment and preprocessing, a pooled QC sample was prepared by combining equal parts of each study sample and analysed periodically among study sample analyses. For UPLC–MS only, a series of QC sample dilutions was created (10 × 100%, 5 × 80%, 3 × 60%, 3 × 40%, 5 × 20%, 10 × 1%) and analysed at the start and end of each set of sample analyses.

NMR and UPLC–MS assays were applied to maximize coverage of a broad range of metabolite classes including lipophilic, hydrophilic, small and macromolecular analytes and processed to include both global profiling and targeted extraction datasets (Table 1). Global profiling provides a comprehensive analysis of all measurable metabolites in a sample but results in datasets with large numbers of variables per analyte, the identities of which are typically unknown. In contrast, by targeted extraction of a predefined set of metabolites, pre-annotated datasets are immediately more interpretable but are limited in coverage to those metabolites in the predefined set.

**Table 1:** Metabolites analysed using each metabolic profiling assay

Technology platform	Metabolic profiling assay	No. features (global profiling datasets)	No. annotated metabolites (targeted extraction datasets)	Metabolome/lipidome coverage	Participants, *n*
UPLC–MS	HILIC+	5729	29	Hydrophilic metabolites including carnitine, betaine, warfarin, caffeine, cotinine, metform, TMAO, proline, creatine, cytosine	SCANS: 83 RUN-DMC: 376 Total: 459
	Lipid RPC−	4336	31	Lipophilic metabolites including bilirubin, fatty acids, lysophosphatic acids, lysophosphocholines, lysophosphoethanolamines	SCANS: 101 RUN-DMC: 447 Total: 548
	Lipid RPC+	7407	190	Lipophilic metabolites including carnitines, cholesteryl esters, ceramides, cholesterol, diglycerides, lysophosphocholines, lysophosphoethanolamines, monoacylglycerols, phosphocholines, phosphethanolamines, sphingomyelins, triglycerides	SCANS: 101 RUN-DMC: 456 Total: 557
NMR	Standard 1D	18 646	14 (IVDr BI-QUANT)	Small molecule metabolites including creatinine, TMAO, alanine, creatine, glutamine, histidine, isoleucine, tyrosine, valine, lactic acid, acetoacetic acid, glucose	SCANS: 115 RUN-DMC: 494 Total: 609
			105 (IVDr BI-LISA)	Lipoprotein subfractions including subtypes of cholesterol, phospholipids, triglycerides and apolipoproteins	SCANS: 105 RUN-DMC: 430 Total: 535
	CPMG	18 646	N/A	Small molecule metabolites	SCANS: 111 RUN-DMC: 451 Total: 562
**Overall**		**54** **764**	**369**		**SCANS: 121** **RUN-DMC: 503** **Total: 624**

CPMG = Carr–Purcell–Meiboom–Gill; IVDr BI-LISA = Bruker IVDr Lipoprotein Subclass Analysis; IVDr BI-QUANT = Bruker IVDr automated quantification of small molecule metabolites; TMAO = trimethylamine-*N*-oxide.

UPLC–MS was applied with two chromatographic techniques: hydrophilic interaction chromatography (HILIC), for the separation of hydrophilic analytes (i.e. polar and charged metabolites) and reverse-phase chromatography (RPC) for the separation of lipophilic analytes (i.e. complex and neutral lipids). When coupled to positive and/or negative mode ionization the following datasets were produced: lipid positive (lipid RPC+), lipid negative (lipid RPC−) and HILIC positive (HILIC+). NMR assays comprised a standard one-dimensional (1D) NMR profile experiment with water presaturation using the 1D-Nuclear Overhauser Effect Spectroscopy presat pulse sequence for characterization of small and macromolecular metabolites and an additional spin-echo experiment using the 1D Carr–Purcell–Meiboom–Gill (CPMG) presat pulse sequence for saturation of macromolecules signals.

For generation of global profiling UPLC–MS datasets, untargeted peak detection was performed using Progenesis QI (Waters Corp.). For targeted extraction, peakPantheR (Peak Picking and ANnoTation of High-resolution Experiments in R)^15^ was used to fit predefined UPLC–MS signals with semi-automated (manually validated) extraction of known chemical species across the three assays. For NMR, targeted extraction was performed using the *in vitro* diagnostics platform (IVDr) from Bruker Biospin (www.bruker.com) generating quantified measurements of both lipoprotein subclasses (BI-LISA) and small molecules (BI-QUANT).

For all datasets, preprocessing and QC was performed using the nPYc-Toolbox^16^ according to previously published criteria.^12,14^ Metabolite intensity values on the lipid RPC+ and lipid RPC− platforms were corrected for run order and batch-related intensity drifts by applying locally estimated scatterplot smoothing (LOESS) regression fitted to the pooled QC samples. Run order and batch correction were not necessary for the metabolites measured on the HILIC+ platform. Only features/metabolites measured with high analytical quality [relative standard deviation (RSD) in pooled QC < 30%, dilution series Pearson correlation to dilution factor >0.7, RSD in study samples >1.1 × RSD in pooled QC] were retained. For the global profiling datasets this resulted in 5729 features for HILIC+, 4336 features for lipid RPC− and 7407 features for lipid RPC+. For the targeted extraction UPLC–MS datasets, a total of 250 unique and known chemical species passed QC across the three assays (29 on HILIC+, 31 on lipid RPC− and 190 on lipid RPC+). For NMR global profiling data, after removal of uninformative spectral regions, 18 646 features were available in both standard 1D and CPMG NMR datasets. Quantification using the Bruker BI-QUANT algorithm resulted in automated quantification of 27 small molecules, of which 14 passed the feature selection criteria; the remaining 13 metabolites were not detected or were not present in sufficient concentrations to be measured accurately. Application of the BI-LISA algorithm resulted in automated quantification of 105 lipoprotein subclasses. Across all assays, discrepancies in final sample numbers available for analysis (Table 1) result from insufficient sample volume for data acquisition, sample compromised during acquisition or sample exclusion owing to data not meeting stringent QC criteria (Supplementary material). To ensure approximately normal distributions, a generalized log transformation was applied to all features/metabolites and the values were rescaled using mean centring and dividing by the standard deviation of each metabolite across participants.

### MRI and clinical end points

Our primary MRI end point was baseline mean diffusivity normalized histogram peak height measured within normal appearing white matter voxels (MDNPH), a DTI marker that has previously been shown to be correlated with, and predictive of, the degree of cognitive impairment.^2,18^ A reduction in MDNPH corresponds with increasing mean diffusivity. Secondary MRI end points that we examined were baseline cerebral microbleed count, lacune count, WMH (expressed as the percentage of WMH volume out of the total brain volume), total brain volume and peak width of skeletonized mean diffusivity (PSMD), an alternative DTI marker that has been shown to be robust and highly sensitive.^19^ Our primary clinical end point was conversion to dementia, which was diagnosed using the fifth edition of the Diagnostic and Statistical Manual of Mental Disorders (DSM-5) definition for major neurocognitive disorder. Secondary clinical end points that we examined consisted of: (i) cognition, assessed by a global cognition score as well as scores for the executive function and processing speed domains; and (ii) disability, assessed by the Barthel index, which is used to measure performance on Activities of Daily Living (ADL). We orientated each outcome so that higher values corresponded to increased cognitive decline (e.g. we analysed brain atrophy as the inverse of total brain volume). We analysed cerebral microbleed and lacune counts both as continuous and binary variables (i.e. presence or absence of microbleeds or lacunes). A further end point that we included was a simple MRI score that accounted for presence of microbleeds, number of lacunes and WMH volume (Fazekas score), which has been shown to improve prediction of dementia in SVD patients.^9^ To obtain comparable effect sizes across outcomes, values for each outcome were rescaled using mean centring and dividing by the standard deviation (SD) across participants. A description of these end points is provided in Supplementary Table 1.

### Statistical analyses

We performed cross-sectional analyses examining the association of baseline MRI markers, cognition and disability data per 1-SD higher metabolite levels measured at baseline. We constructed linear regression models for continuous outcomes and logistic regression models for binary outcomes, with adjustment for cohort, baseline age and sex. We also conducted these analyses with further adjustment for diabetes status, hypertension status and hypercholesterolaemia status to determine whether the associations of metabolites with imaging markers and cognition were modified by relevant risk factors.

To evaluate the relationship of metabolites with changes in MRI parameters and cognition over time, we calculated an annualized change in values for each outcome on the basis of the difference in values between the baseline and latest time point divided by the amount of follow-up time that had elapsed. We then ran linear regression models examining the association of annualized change in MRI markers, cognition and disability per 1-SD higher metabolite levels.

We also performed longitudinal analyses to determine whether metabolites measured at baseline predict long-term conversion to dementia, for which we constructed Cox proportional hazards regression models adjusted for cohort, age and sex to assess the association of conversion to dementia per 1-SD higher metabolite levels.

Analyses were conducted using R v.4.1.1 (R Core Team, 2021). To account for multiple testing comparisons, we used a false discovery rate (FDR) threshold of *q* < 0.05 to identify significant associations for each outcome measure. Two-sided *P*-values and 95% confidence intervals are presented.

### Sensitivity analyses

We conducted several sensitivity analyses to further examine the independent nature of the associations. First, we conducted analyses at baseline separately within each cohort for significantly associated metabolites to compare the magnitude and direction of associations across cohorts. Second, we conducted analyses stratified by age to compare associations of metabolites with MRI markers and cognition in younger (<65) and older (≥65) populations. Third, we conducted a subgroup analysis in participants with scores of ≥2 on the Fazekas scale to evaluate whether there were any differences in the magnitude of the associations in individuals with more severe forms of SVD.

### Data availability

The raw metabolomics data described in this study were generated at the Medical Research Council National Institute for Health Research (MRC-NIHR) National Phenome Centre. Derived data supporting the findings of this study are available from the corresponding author on request.

## Results

### Patient characteristics

In this study, we analysed individual participant data from 624 patients with symptomatic SVD. Most participants were male (58%) and white (91%), with a mean (SD) age of 66.5 (9.2) years (Supplementary Table 2). Compared to participants from RUN-DMC, SCANS participants were on average 4.4 years older, came from more diverse ethnic backgrounds (23% Caribbean and 6% African in SCANS), had higher rates of hypertension and hypercholesterolaemia and had more severe SVD as indicated by increased WMH volume.

### Associations with baseline imaging parameters

We obtained measurements for 369 annotated metabolites and lipoprotein subclasses measured on five different metabolomics platforms that used both UPLC–MS and NMR (Table 1). We analysed the association of these metabolites with MDNPH and conversion to dementia, as well as with a range of MRI markers and indicators of cognition and disability (Supplementary Table 1).

**Figure 1:**
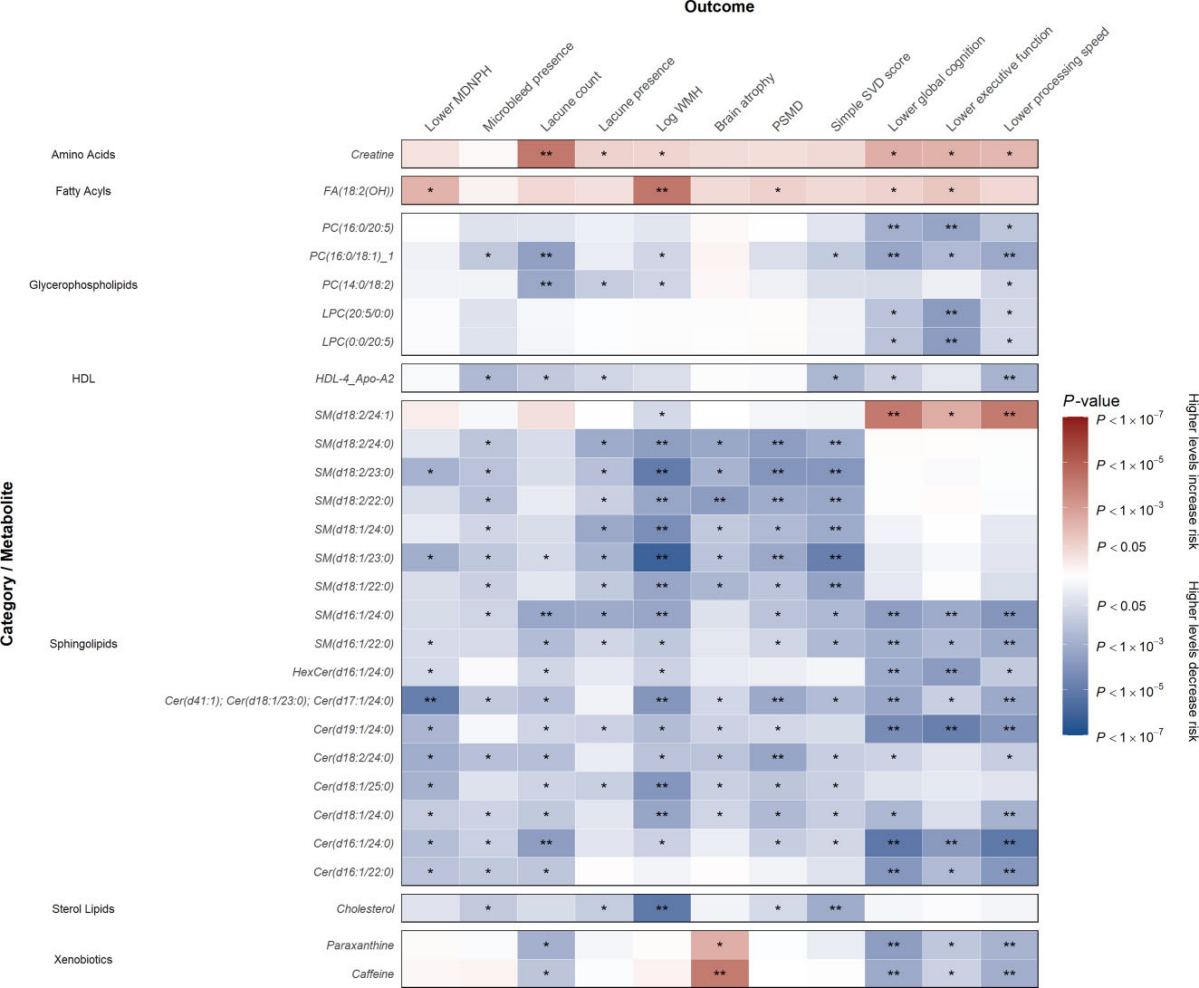
**Association of MRI markers and cognition parameters at baseline per 1-SD higher metabolite levels with further adjustment for relevant risk factors.** Beta estimates and *P*-values were obtained from linear or logistic regression models adjusted for cohort, baseline age, sex, diabetes status, hypertension status and hypercholesterolaemia status. Colours show magnitude and direction of *P*-value for association of metabolite with each outcome (red indicates positive association and blue indicates inverse association). Asterisks indicate significance: *P < 0.05; **FDR *q* < 0.05.

In cross-sectional analyses adjusted for relevant demographic (cohort, baseline age and sex) and vascular risk factors (diabetes, hypertension and hypercholesterolaemia status), 28 metabolites were associated with baseline imaging parameters and cognition indicative of increased SVD load (Fig. 1, Table 2 and Supplementary Table 3). Higher levels of creatine, FA(18:2(OH)) and SM(d18:2/24:1) were associated with higher lacune count and WMH volume and impaired cognition. Conversely, lower levels of glycerophospholipids (*n* = 5), sphingolipids (*n* = 16), HDL-4_ApoA2 and cholesterol were associated with reduced SVD load (i.e. lower MDNPH, lacune count, WMH volume, PSMD and improved cognition).

**Table 2:** Summary of associations of metabolites with MRI/DTI markers and cognition parameters at baseline with adjustment for vascular risk factors

Metabolite category/name		Association with MRI/DTI markers	Association with cognition
**Amino acids**			
Creatine		Lacune count** Lacune presence* log WMH*	Lower global cognition* Lower executive function* Lower processing speed*
**Fatty acyls**			
Unsaturated fatty acids: FA (18:2(OH))		Lower MDNPH* log WMH** PSMD*	Lower global cognition* Lower executive function*
**Glycerophospholipids**			
Diacylglycerophosphocholines: PC (16:0/20:5), PC(16:0/18:1)_1, PC (14:0/18:2)		(Microbleed presence*) (Lacune count**) (Lacune presence*) (log WMH*)	(Lower global cognition**) (Lower executive function**) (Lower processing speed**)
Monoacylglycerophosphocholines: LPC (20:5/0:0), LPC (0:0/20:5)			(Lower global cognition*) (Lower executive function**) (Lower processing speed*)
**Sphingolipids**			
Ceramide phosphocholines (sphingomyelins):	SM(d18:2/24:0), SM(d18:2/23:0), SM(d18:2/22:0), SM(d18:1/24:0), SM(d18:1/23:0), SM(d18:1/22:0), SM(d16:1/24:0), SM(d16:1/22:0)	(Lower MDNPH*) (Microbleed presence*) (Lacune count**) (Lacune presence*) (log WMH**) (Brain atrophy**) (PSMD**)	(Lower global cognition**) (Lower executive function**) (Lower processing speed**)
	SM(d18:2/24:1)	(log WMH*)	Lower global cognition** Lower executive function* Lower processing speed**
Hexosylceramides: HexCer(d16:1/24:0)		(Lower MDNPH*) (Lacune count*) (log WMH*)	(Lower global cognition**) (Lower executive function**) (Lower processing speed*)
N-acylsphingosines (ceramides): Cer(d41:1)/Cer(d18:1/23:0)/Cer(d17:1/24:0), Cer(d19:1/24:0), Cer(d18:2/24:0), Cer(d18:1/25:0), Cer(d18:1/24:0), Cer(d16:1/24:0), Cer(d16:1/22:0)		(Lower MDNPH**) (Microbleed presence*) (Lacune count**) (Lacune presence*) (log WMH**) (Brain atrophy*) (PSMD**)	(Lower global cognition**) (Lower executive function**) (Lower processing speed**)
**Sterol lipids**			
Cholesterol		(Microbleed presence*) (Lacune presence*) (log WMH**) (PSMD*)	
**Xenobiotics**			
Paraxanthine		(Lacune count*) Brain atrophy*	(Lower global cognition**) (Lower executive function*) (Lower processing speed**)
Caffeine		(Lacune count*) Brain atrophy**	(Lower global cognition**) (Lower executive function*) (Lower processing speed**)
**HDL**			
Analytes within HDL: HDL-4_Apo-A2		(Microbleed presence*) (Lacune count*) (Lacune presence*)	(Lower global cognition*) (Lower processing speed**)

Associations were obtained from linear or logistic regression models adjusted for cohort, baseline age, sex, diabetes status, hypertension status and hypercholesterolaemia status. Asterisks indicate significance: *P < 0.05; **FDR *q* < 0.05. Inverse associations of metabolites with MRI/DTI markers and cognition are enclosed in parentheses to indicate negative beta coefficients.

In analyses adjusted for demographic factors alone, lower serum levels of 34 sphingolipids (including sphingomyelins and ceramides) were associated with lower MDNPH, higher WMH volume, greater brain atrophy and impaired cognition (Supplementary Fig. 1A and Supplementary Table 4). Lower levels of 30 glycerophospholipids (including phosphatidylcholines and lysophosphatidylcholines) were also associated with lower MDNPH, greater brain atrophy and impaired cognition. Higher levels of seven amino acids and nucleotides (*N*^1^-acetylspermidine, *N*-acetylputrescine, isoleucine, creatinine, creatine, cytosine and 5′-methylthioadenosine) were associated with lower MDNPH, higher WMH volume and greater brain atrophy (Supplementary Fig. 1B and Supplementary Table 4). Lower levels of bilirubin were associated with impaired cognition. Higher levels of caffeine were associated with greater brain atrophy but also with improved cognition.

### Longitudinal analyses of progression of MRI parameters and cognition and of incident dementia

**Figure 2:**
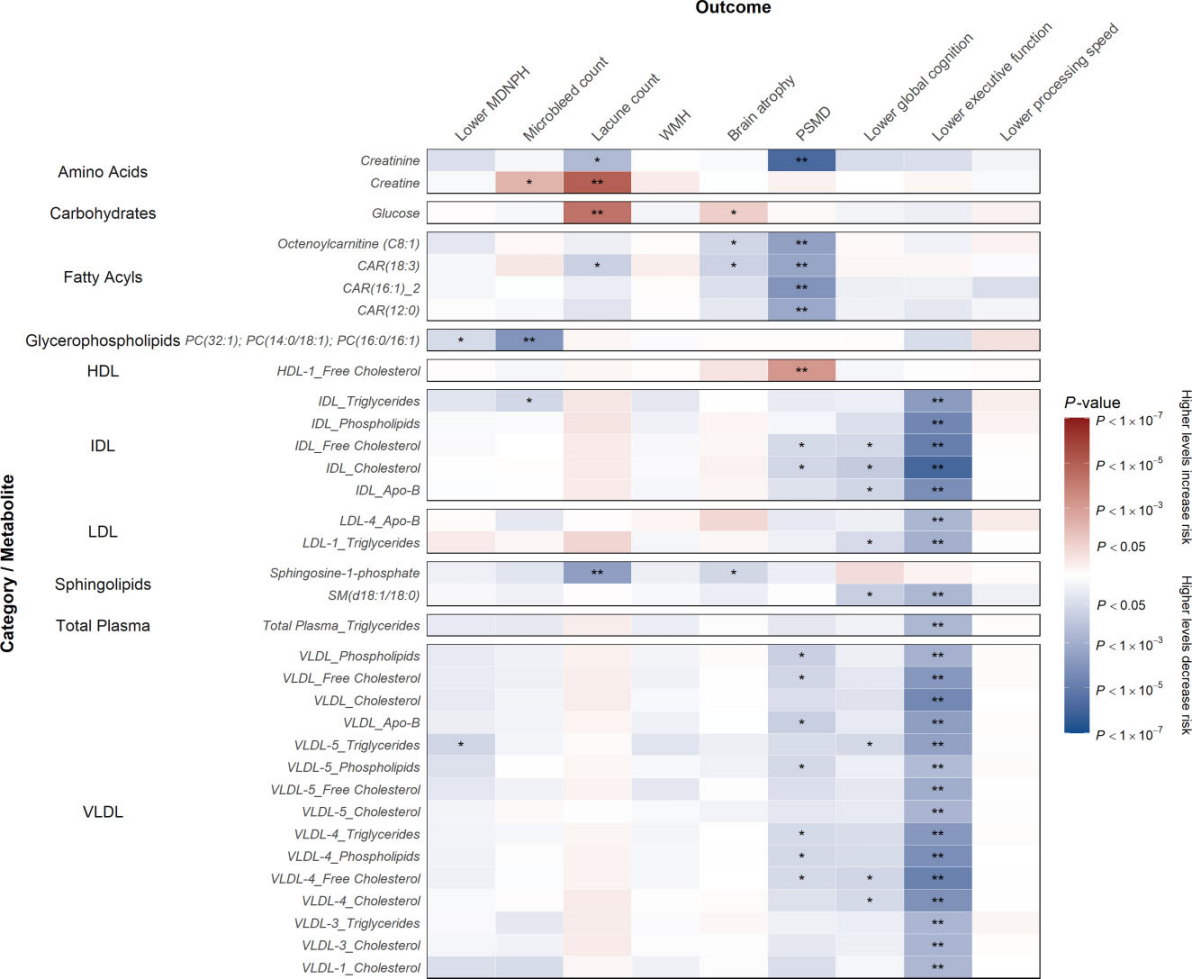
**Association of annualized change in MRI markers and cognition parameters per 1-SD higher metabolite levels.** Annualized changes in outcomes were calculated as the differences in values between the baseline and latest time points divided by the amount of follow-up time. Beta estimates and *P*-values were obtained from linear or logistic regression models adjusted for baseline age, sex and cohort. Colours show magnitude and direction of *P*-value for association of metabolite with each outcome (red indicates positive association and blue indicates inverse association). Asterisks indicate significance: *P < 0.05; **FDR *q* < 0.05.

We also analysed the association of metabolites at baseline with annualized change in levels of imaging markers and cognition (Fig. 2 and Supplementary Table 5). Lower levels of four carnitines and creatinine were associated with higher annualized change in PSMD, and lower levels of 23 lipoprotein analytes in intermediate-density (IDL), low-density (LDL) and very low-density lipoprotein cholesterol (VLDL) and total plasma were associated with higher annualized change in impaired executive function. Higher levels of creatine and glucose were associated with increased annualized change in number of lacunes.

**Figure 3:**
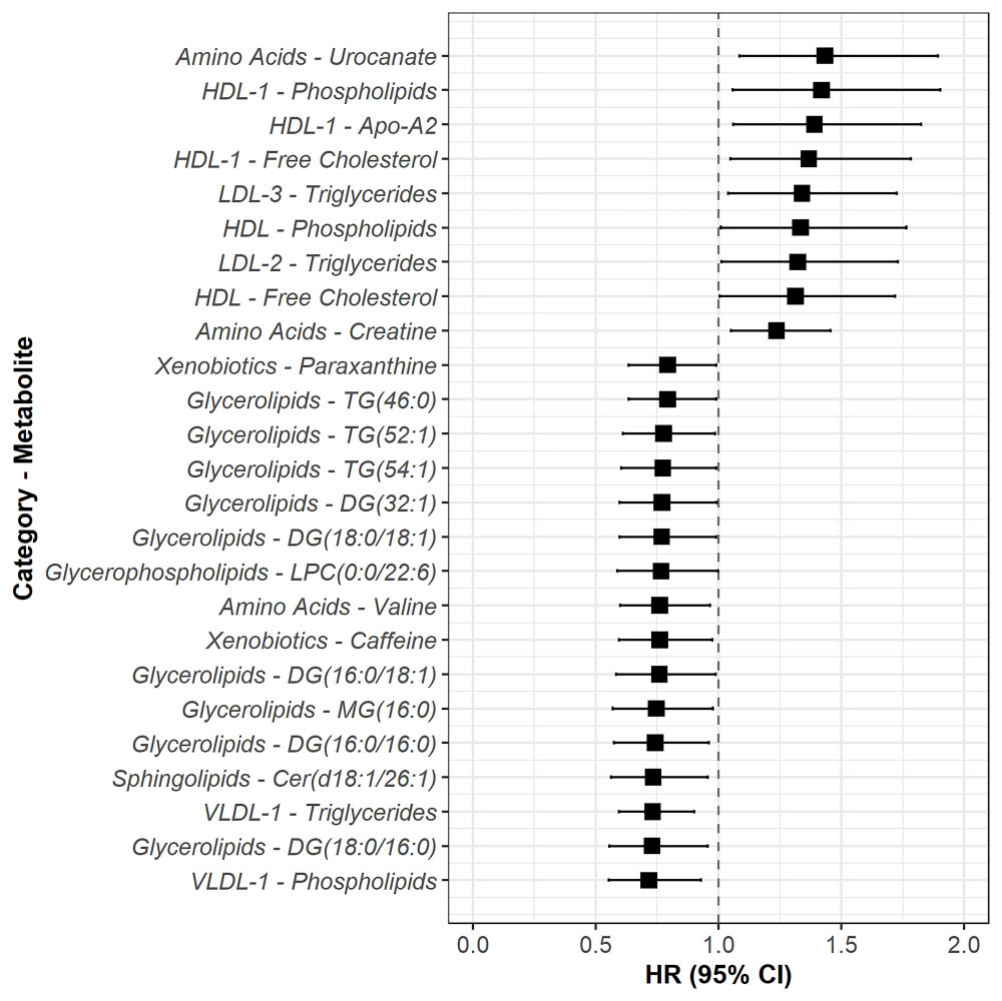
**Adjusted hazard ratios for dementia per 1-SD higher metabolite levels.** Analyses were adjusted for baseline age, sex and cohort. Filled squares indicate associations that were significant at *P* < 0.05.

When accounting for long-term follow-up in time-to-event analyses, future incidence of dementia was associated with 25 metabolites, including lower levels of valine, caffeine and VLDL analytes, and higher levels of urocanate, lipoprotein analytes in high-density lipoprotein cholesterol (HDL) and LDL, and creatine (Fig. 3 and Supplementary Table 6). These associations were suggestive (*P* < 0.05) but not statistically significant (FDR *q* < 0.05) after correcting for multiple testing.

### Sensitivity analyses

Analyses conducted separately within each cohort showed that the directions of association were mostly consistent between SCANS and RUN-DMC, but there were some differences in the magnitudes of the associations (Supplementary Fig. 2 and Supplementary Table 7). Lower levels of multiple glycerolipids (triglycerides and diglycerides) were associated with lower MDNPH and impaired cognition in SCANS participants, with no evidence of an association in RUN-DMC participants. Lower levels of multiple glycerophospholipids and sphingolipids were associated with lower MDNPH and impaired cognition in both SCANS and RUN-DMC, but the specific lipids that reached statistical significance within each lipid class varied. Lower levels of many of these sphingolipids were also associated with increased WMH volume, greater atrophy and higher PSMD in RUN-DMC participants, but not in SCANS participants.

Analyses stratified by age showed stronger associations of metabolites with imaging markers and cognition in older individuals (Supplementary Fig. 3 and Supplementary Table 8), though the substantial reduction in power resulted in fewer significantly associated metabolites overall. We found that four sphingomyelins [SM(d18:2/24:0), SM(d18:2/23:0), SM(d18:1/24:0), and SM(d18:1/23:0)] were inversely associated with the presence of lacunes and WMH volume in older individuals, whereas these associations were all attenuated in younger individuals. Additionally, caffeine was significantly associated with brain atrophy in older individuals but there was no significant association in younger individuals. Many of the other metabolites from the 28 that were statistically significant in the overall analysis adjusted for vascular risk factors were no longer significant in the analyses stratified by age after correction for multiple testing.

An analysis of individuals with more severe forms of SVD, as indicated by scores of ≥2 on the Fazekas scale, did not result in any metabolites that were significantly associated with imaging markers or cognition after FDR correction, which is probably due to the substantial decrease in power (Supplementary Fig. 4 and Supplementary Table 9). The strongest associations, which were still significant at *P* < 0.05, were with microbleeds, lacunes and the simple SVD score, which is not surprising since the Fazekas score (a grading of the volume of WMH) is one of the criteria that are used to calculate the SVD score alongside microbleed count and lacune count.

### Analyses of global profiling datasets

To provide a more global overview, in addition to analyses conducted on the targeted extraction datasets, analyses were also conducted on the unannotated, global profiling datasets. These analyses also revealed statistically significant associations with a number of features. From a total of 54 764 measured features, after correcting for multiple testing using an FDR threshold of *q* < 0.05, we identified 1362 features associated with lower MDNPH, 2474 features associated with increased WMH volume and 1533 features associated with executive function (Supplementary Tables 10 and 11). Despite the larger number of features measured using NMR, a greater proportion of the significant associations were with features derived from UPLC–MS datasets, resulting from increased depth of coverage (UPLC–MS assays weighted to lipids, NMR weighted to small molecules) and the higher degree of redundancy in the NMR global profiling data (multiple features derived from the same underlying metabolite). Similar to the analyses conducted in the smaller set of annotated metabolites, there were no significant associations of features with microbleed count or conversion to dementia in the global profiling datasets.

## Discussion

In this comprehensive metabolomics profiling study of over 600 individuals with MRI-confirmed SVD, we identified 28 metabolites (creatine, an unsaturated fatty acid, five glycerophospholipids, 17 sphingolipids, cholesterol, paraxanthine, caffeine and a lipoprotein) that are significantly associated with SVD imaging markers and cognition, and 25 metabolites (lipoprotein subclasses, amino acids and xenobiotics) that are significantly associated with progression to dementia. We found that decreased levels of multiple glycerophospholipids, sphingolipids and sterol lipids are associated with increased SVD load as evidenced by higher WMH volume, lower MDNPH and greater atrophy, as well as with impaired cognition. We also found that higher levels of creatine, FA(18:2(OH)) and SM(d18:2/24:1) are associated with higher lacune count, WMH volume and cognition.

The associations with glycerophospholipids and sphingolipids were particularly notable. Previous metabolomics studies have shown associations of lower levels of ceramide ratios with fewer number of cerebral microbleeds^20^ and increased risk of incident dementia,^21^ and another study showed associations of ceramides and sphingomyelins with SVD.^22^ In the present study, lower levels of serum sphingomyelins and ceramides were associated with lower MDNPH, higher WMH volume and PSMD, increased number of lacunes, greater brain atrophy and impaired cognition in baseline analyses even after further adjustment. Only one sphingomyelin had a statistically significant association with executive function when assessing the annualized change in metabolite levels, suggesting that the absolute levels of the metabolites at baseline are more relevant in evaluating their effects on SVD and cognition than how those levels change over time.

Demyelinating diseases such as multiple sclerosis cause neuroinflammation, which can result in damage to the myelin sheath. Inflammation has also been proposed to play a role in the progression of SVD,^23^ and metabolites could be implicated in the causal pathway. Previous studies have shown that patients with multiple sclerosis and other demyelinating diseases have increased levels of sphingomyelins and ceramides in CSF.^24,25^ However, these ceramides and sphingomyelins have also been implicated in non-neurological conditions such as heart failure,^26^ so further research is needed to disentangle these associations and better understand the underlying pathophysiology.

Several magnetic resonance spectroscopy studies of acute stroke patients have reported reduced levels of creatine in areas of cerebral infarction.^27,28^ However, in our study we found that increased levels of creatine in circulating serum were associated with increased number of lacunes and WMH volume, cognitive impairment and increased risk of incident dementia. One possible explanation is that SVD damage causes the release of creatine through damage to neurons or glial cells, which is therefore depleted in the brain and increased in the circulating blood.

Linoleic acid [FA(18:2(OH))] is an essential omega-6 fatty acid obtained from plant sources. Diets rich in linoleic acid and other omega-6 fatty acids inhibit the metabolic formation of omega-3 polyunsaturated fatty acids, which can lead to a deficit of eicosapentaenoic acid (EPA)^29^ and is associated with reduced brain volume, impaired cognition and accelerated progression to dementia.^30^ Our study showed that increased levels of linoleic acid were associated with lower MDNPH, higher WMH volume and PSMD, and impaired cognition.

We observed a significant inverse association of cholesterol with WMH volume. A previous study employing Mendelian randomization demonstrated evidence that genetically elevated levels of HDL-C are associated with lower WMH volume and lower risk of SVD,^31^ confirming our findings.

Another finding from our study was that increased caffeine consumption (i.e. higher levels of caffeine, and its primary metabolite paraxanthine) was associated with lower total brain volume but improved cognition, particularly processing speed and decreased risk of dementia. Numerous systematic reviews have demonstrated the positive benefits of caffeine consumption,^32^ but studies have also shown that coffee consumption is associated with increased risk of Alzheimer's disease,^33,34^ although there is no evidence of a causal relationship of coffee consumption with SVD or other ischaemic stroke subtypes.^35^ One explanation for our findings is that caffeine can be associated with short-term improvement in cognitive functioning but that long-term consumption is associated with chronic brain atrophy.

We found that VLDL analytes were associated with lower risk of incident dementia, which confirms findings from a previous analysis of eight prospective cohort studies, which found that increased levels of VLDL lipoprotein subclasses were associated with lower risk of dementia.^36^

Our findings have several important clinical implications. First, they may provide novel insights into pathogenic mechanisms underlying SVD; it is possible that modifying levels of specific metabolites could help reduce the risk of cognitive decline and dementia in patients with SVD. Dietary interventions or novel therapies could improve long-term outcomes for SVD patients. Second, a metabolomics panel based on these associations could be developed for clinicians to predict those patients who are most likely to progress to more severe forms of dementia and offer personalized treatment. Third, exploration of the broader metabolic profiles derived from our investigation show promise for the discovery and identification of additional markers yielding greater mechanistic insight to the relevant phenotypes.

A key issue is whether metabolites predict future risk, rather than merely cross-sectionally associations with markers of SVD (i.e. whether the associations are causal). A major strength of our study is that we also included prospective longitudinal data to determine whether metabolites at baseline predicted future dementia risk. This provides some evidence supporting causality. It would be beneficial to conduct further studies with a longer follow-up period and a larger sample size to help address this question more fully. Other approaches such as Mendelian randomization, which uses genetic variants as instrumental variables in an approach akin to a randomized trial,^37^ would be useful to assess causality, but we lacked sufficient power in this study to perform these analyses.

The strengths of our study include the fact that the metabolites, indicators of cognitive function and brain MRI markers were measured together at baseline, with MRI and cognitive data also available at multiple timepoints, and with long-term prospective follow-up of 5–14 years. Second, the metabolites were measured using a robust, highly accurate, validated analytical approach with QC measures. Third, we conducted sensitivity analyses separately within each cohort as well as stratified by age and in a subset of individuals with more severe forms of SVD. Although the magnitude of the associations attenuated in these subgroup analyses, this may have been simply due to loss of power. Fourth, to examine whether adjustment for vascular risk factors may have increased bias or decreased the level of precision in the estimates, we also conducted analyses adjusted only for baseline demographic factors.

The differences in the magnitudes of the associations between cohorts is probably because RUN-DMC had a larger population and a wider range of disease, whereas SCANS was a much smaller population and was more homogeneous, with all patients having moderate or severe SVD on MRI. MRI features of SVD can coexist with other stroke pathologies.^38^ In SCANS, strict exclusion criteria were used to exclude large artery stenosis and cardioembolic sources of stroke, whereas in RUN-DMC only MRI criteria were applied and coexistent large artery atheromatous disease was not excluded.

Our study also has limitations. First, even though this is one of the largest metabolomics studies on SVD so far, the sample sizes of the studies were still modest, which reduced the power to detect associations and prevented us from fully addressing the question of causality. Second, the large number of statistical tests conducted meant that some associations may have been biologically and clinically meaningful but did not reach the threshold for statistical significance after correction for multiple testing. However, we applied an FDR correction to reduce the likelihood of identifying false positives. Third, the metabolites we identified could be on the causal pathway for cognitive decline or dementia, but secondary to tissue damage caused by demyelination.^39^ We were unable to evaluate this because patients in SCANS were not assessed for multiple sclerosis and myelin loss was not measured, so further mechanistic and longitudinal studies are needed. However, even if changes in metabolite levels do not directly cause cognitive decline or dementia, they could still be useful predictors of these conditions. Fourth, the metabolites were measured in blood serum rather than CSF, which is considered better suited to measurement of sensitive biomarkers of neurological and cognitive decline,^40^ although studies have shown similar changes in affected pathways for metabolites measured in blood and CSF.^41^ However, serum biomarkers are clinically useful as serum is much less invasive to collect from patients. Fifth, we did not examine ratios of metabolites, which can reveal additional insights into metabolic pathways^42^ and should be examined in follow-up analyses. Sixth, two different methods were used to analyse the DTA data. PSMD and MDNPH are both DTI markers used to quantify mean diffusivity from the DTI scan, but they calculate it in different ways. PSMD is calculated from the DTI skeleton using track-based spatial statistics rather than the entirety of the white matter, as is the case for MDNPH. The differences in how PSMD and MDNPH were calculated could explain some of the differences in the associations that were observed for these DTI markers. Finally, the study was conducted in patient populations with symptomatic SVD and may not be generalizable to other contexts.

In conclusion, we provide consistent evidence that multiple serum metabolites are associated with SVD severity on MRI, cognitive decline and incident dementia in patients with cerebral SVD. Further research should be conducted to identify whether these associations are causal and could be used to improve the ability of clinicians to predict the rate of progression and severity of onset of lacunar stroke and dementia, and for researchers to develop novel treatment approaches for patients at increased risk of these conditions.

## Supplementary Material

awac041_Supplementary_Data

## Abbreviations

DTI: diffusion tensor imaging
MDNPH: mean diffusivity normalized histogram peak height measured within normal appearing white matter voxels
NMR: nuclear magnetic resonance
UPLC–MS: ultra-performance liquid chromatography–mass spectrometry
PSMD: peak width of skeletonized mean diffusivity
RUN-DMC: Radboud University Nijmegen Diffusion Tensor and Magnetic Resonance Imaging Cohort
SCANS: St George's Cognition and Neuroimaging in Stroke
SVD: small vessel disease
WMH: white matter hyperintensities
